# An essential gene signature of breast cancer metastasis reveals targetable pathways

**DOI:** 10.1186/s13058-024-01855-0

**Published:** 2024-06-12

**Authors:** Yiqun Zhang, Fengju Chen, Marija Balic, Chad J. Creighton

**Affiliations:** 1 Dan L. Duncan Comprehensive Cancer Center, Baylor College of Medicine, One Baylor Plaza, MS305, Houston, TX 77030 USA; 2 Division of Oncology, Department of Internal Medicine, Medical University of Graz, Graz, Austria; 3 Unit for Translational Breast Cancer Research, Medical University of Graz, Graz, Austria; 4 Division of Hematology/Oncology, Department of Medicine, University of Pittsburgh, Pittsburgh, PA USA; 5 Human Genome Sequencing Center, Baylor College of Medicine, Houston, TX 77030 USA; 6 Department of Medicine, Baylor College of Medicine, Houston, TX USA

## Abstract

**Background:**

The differential gene expression profile of metastatic versus primary breast tumors represents an avenue for discovering new or underappreciated pathways underscoring processes of metastasis. However, as tumor biopsy samples are a mixture of cancer and non-cancer cells, most differentially expressed genes in metastases would represent confounders involving sample biopsy site rather than cancer cell biology.

**Methods:**

By paired analysis, we defined a top set of differentially expressed genes in breast cancer metastasis versus primary tumors using an RNA-sequencing dataset of 152 patients from The Breast International Group Aiming to Understand the Molecular Aberrations dataset (BIG-AURORA). To filter the genes higher in metastasis for genes essential for breast cancer proliferation, we incorporated CRISPR-based data from breast cancer cell lines.

**Results:**

A significant fraction of genes with higher expression in metastasis versus paired primary were essential by CRISPR. These 264 genes represented an essential signature of breast cancer metastasis. In contrast, nonessential metastasis genes largely involved tumor biopsy site. The essential signature predicted breast cancer patient outcome based on primary tumor expression patterns. Pathways underlying the essential signature included proteasome degradation, the electron transport chain, oxidative phosphorylation, and cancer metabolic reprogramming. Transcription factors MYC, MAX, HDAC3, and HCFC1 each bound significant fractions of essential genes.

**Conclusions:**

Associations involving the essential gene signature of breast cancer metastasis indicate true biological changes intrinsic to cancer cells, with important implications for applying existing therapies or developing alternate therapeutic approaches.

**Supplementary Information:**

The online version contains supplementary material available at 10.1186/s13058-024-01855-0.

## Introduction

Breast cancer metastasis is a complicated and poorly understood process for which there is a shortage of effective treatments [1]. A better understanding of the mechanisms of metastasis could eventually lead to more effective treatment of the disease, greatly extending patient life or even curing patients [2, 3]. New insights into breast cancer metastasis could be obtained by molecular profiling of both metastatic and primary breast cancers [4–11]. Genes differentially altered in sequence or expression might provide clues as to the pathways or processes underlying metastasis. At the DNA level, except for *ESR1* mutations, almost no recurrent mutations are unique to metastatic compared to primary breast cancers [4, 5]. However, a higher tumor mutational burden has been observed in metastatic samples compared to the paired primary samples [4]. At the gene expression level, widespread differences may distinguish a metastatic tumor from its primary tumor pair from the same patient [4–11]. Some of these differences might represent changes intrinsic to the cancer cells, including instances of molecular subtype switching [4–6, 12]. Other expression differences would be extrinsic to cancer cells and might involve concerted changes in the tumor microenvironment relevant to the metastatic process [13] or may simply reflect differences in the tissue and cell composition of the respective tumor samples [14].

While the differential expression profile of breast cancer metastases represents an avenue for discovering new or underappreciated pathways for therapeutic targeting, a notable challenge would work against our ability to utilize such data optimally. As a tumor biopsy sample is comprised of multiple non-cancer cell types—including fibroblasts, immune cells, endothelial cells, normal epithelial cells, and differentiated cells specific to the site of biopsy—distinguishing cancer-specific from non-cancer-specific differential patterns within an aggregate expression profile is non-trivial [6, 15]. Comparing bulk expression profiles for primary versus metastatic samples would largely involve comparisons between breast and non-breast tissues, respectively, particularly as the metastases may be sampled at distal sites from the breast. Such tissue-specific expression differences represent artifacts rather than actual metastasis biology. As opposed to bulk RNA-sequencing (RNA-seq), single-cell RNA sequencing (scRNA-seq) might be one potential avenue for discerning cancer cell-specific patterns, but to date, no scRNA-seq studies have profiled appreciable numbers of breast cancer metastases and paired primaries [16]. In addition, most genes in practice may not be covered sufficiently by the scRNA-seq platform. True positive genes representing tumor biology may be present within the differential profile, though these might represent just a fraction of the hundreds or even thousands of genes that would appear differentially expressed. Incorporating outside orthogonal data can help distinguish from the global molecular profile the cancer cell-intrinsic genes relevant to breast cancer metastasis.

This present study aimed to define a gene expression signature of breast cancer metastasis versus paired primaries, for which the genes would be essential to cancer cells. The Breast International Group (BIG) conducted Aiming to Understand the Molecular Aberrations in Metastatic Breast Cancer (AURORA), a molecular screening initiative that recently published a gene expression profiling dataset of 152 breast cancer patients, including both a metastasis and paired primary sample for each patient [4]. To help sift through the top differential metastasis genes from the BIG-AURORA dataset, we integrated data from CRISPR assays in breast cancer cell lines [17]. A significant fraction of genes with higher expression in breast cancer metastases versus paired primary were essential for breast cancer cell proliferation by CRISPR. This “essential” metastasis signature was entirely distinct from nonessential metastasis genes, the latter largely representing differences in tissue and cell composition between primary and metastatic biopsy sites. We could also characterize the essential gene signature of breast metastasis regarding associated pathways and transcription factors.

## Results

### A gene expression signature of breast cancer metastasis versus paired primaries

**Fig. 1:**
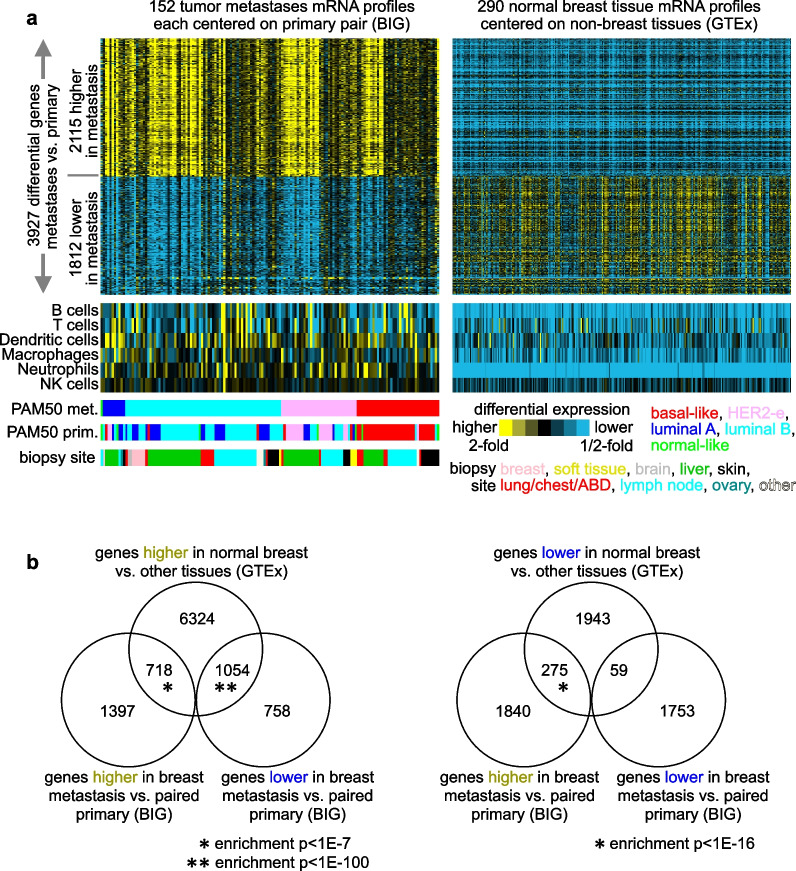
A gene expression signature of breast cancer metastases versus paired primary largely reflects non-breast versus breast tissue differences. **a** Heat map of differential expression in metastasis versus paired primary for a set of 3929 differentially expressed genes with *p* < 0.001 (paired t-test using log2-transformed data). Each metastasis expression profile was centered on its primary pair (not shown). Breast cancer expression data involving 152 metastases with paired primary are from the Breast International Group (BIG) [4]. Yellow, high expression in metastasis versus primary; blue, low expression in metastasis. Alongside the differential genes from the breast cancer dataset are the corresponding differential expression patterns of normal breast tissues (relative to non-breast tissues) from the GTEx dataset [19]. Heat maps of gene expression-based signatures of immune cell infiltrates [20] (taking the average log2 fold change from paired primary for the immune cell type marker genes) are also shown (NK cells natural killer cells). **b** Venn diagrams showing the overlapping genes between BIG breast cancer metastasis and GTEx normal breast (respectively considering high and low gene lists from each dataset). Breast cancer metastasis genes are from part a. GTEx normal breast genes by *p* < 0.000001, comparing log2 expression between normal breast and other normal tissues. Enrichment *p *values by chi-square test. From parts a-b, the observed global associations of the differential metastasis versus primary genes with both tumor biopsy site and GTEx breast expression patterns indicate that most of the observed expression differences in metastases would involve differences in non-cancer cells between the metastasis biopsy site and the breast, respectively, rather than representing changes intrinsic to cancer cells

The BIG AURORA RNA-sequencing (RNA-seq) dataset [4] of 152 breast cancer metastases with paired primaries (from the same patient) represented an opportunity for us to explore gene expression differences occurring in metastases versus primary tumors across so many patient tumors. In contrast to an unpaired analysis, the paired analysis would identify consistent differences between metastasis and primary occurring within the same patient, as the corresponding primary provides a baseline. Widespread differences between metastasis and primary by paired analysis were identified (Data File S1). At a significance level of *p* < 0.001 (paired t-test using log2-transformed values), 3929 genes were differential out of 28,248 uniquely identified genes represented in the dataset. A visual inspection of the differential patterns by heatmap (Fig. 1a) showed these to transcend the PAM50 intrinsic molecular subtype [18] assignments of the metastasis or primary sample, notwithstanding instances of molecular subtype switching [4, 5, 12].

However, we did find a clear indication that most of the observed expression differences between metastases and primary tumors would involve differences in non-cancer cells between the metastasis biopsy site and the breast, respectively, rather than representing changes intrinsic to cancer cells. We arrived at this conclusion from two observations. Firstly, we noted that the relative intensities of the differential patterns (higher versus lower fold changes) in each metastasis sample compared to its corresponding primary pair tracked closely with the metastasis biopsy site (Fig. 1a). Those metastases sampled from the liver had higher or lower fold differences (for genes statistically higher versus lower in metastasis, respectively) than metastases from other sites. Indeed, the genes with the highest fold changes at *p* < 0.001 included many that could be attributed to liver-specific functions, such as genes encoding albumin, apolipoproteins, and fibrinogens (Data File S1). Secondly, we examined the metastasis expression signature (from the BIG dataset) alongside the corresponding differential expression patterns of normal breast tissues relative to non-breast tissues, using the GTEx dataset [19]. Most genes significantly higher in breast cancer metastasis were lower in normal breast versus non-breast tissues, and vice versa (Figs. 1a and b and Data File S1). The expression profile of biopsied tumor samples would include the cells surrounding the tumor as well as the cancer cells themselves, which represented a confounding factor in our analyses. Analysis of gene expression signatures of immune cell types [20] indicated that overall levels of T cell and B cell infiltrates were statistically lower on average in metastases versus its paired primary (*p* < 0.01, paired t-test, Fig. 1a), though these immune cell types often appeared higher in lymph node biopsies [4, 13].

### An essential gene expression signature of breast cancer metastases

**Fig. 2:**
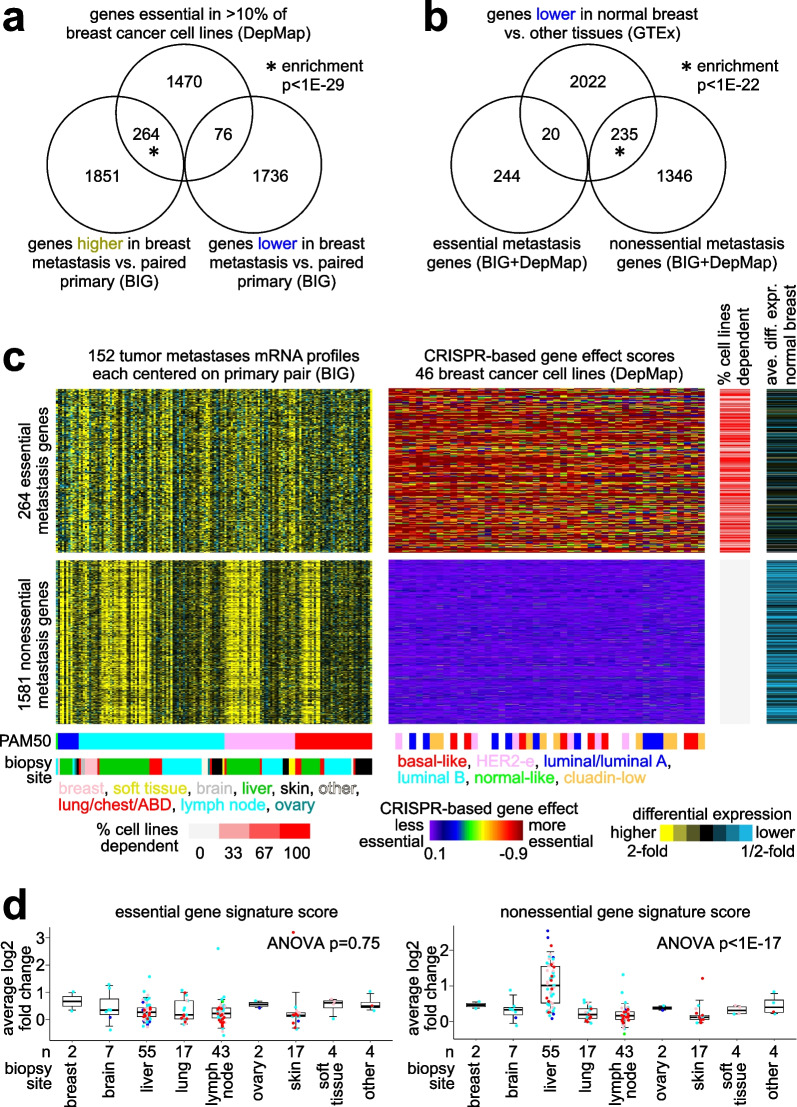
An essential gene expression signature of breast cancer metastases versus paired primary. **a** Venn diagram showing the overlapping genes between the genes essential in > 10% of breast cancer cell lines, according to the DepMap dataset [17, 21] (using CRISPR-based gene effect score < − 0.75 to call essentiality for a given gene and cell line), and the metastasis signature genes (from Fig. 1a). Enrichment p-value by chi-square test. The 264 genes, both essential by DepMap and higher in expression in metastasis versus primary, represent an essential metastasis signature. **b** Venn diagram showing the overlaps between genes lower in normal breast tissue (by GTEx) and either the 264 essential signature genes (part a) or the 1581 “nonessential” genes (higher in metastasis but with no breast cell lines having scores < − 0.75). Enrichment p-value by chi-square test. **c** Heat maps of differential expression in metastasis versus paired primary for essential and nonessential metastasis signature genes (top and bottom, respectively, from parts a and b). Alongside the differential genes from the breast cancer BIG dataset are the corresponding CRISPR-based gene effect scores (from DepMap) and the average differential expression in normal breast versus other normal tissues (from GTEx). **d** By metastasis tissue biopsy site, average log2 fold change in expression between the breast cancer metastases and their paired primaries, represented separately for the essential metastasis genes (left) and the nonessential metastasis genes (right). Box plots represent 5% (lower whisker), 25% (lower box), 50% (median), 75% (upper box), and 95% (upper whisker). Data points are colored according to the PAM50 subtype of the metastasis. From parts b-d, we observe that the issues involving the contribution of non-cancer cells to the differential metastasis expression profile (e.g., as highlighted in Fig. 1) are not present in the essential metastasis signature

We hypothesized that a fraction of genes with differential patterns in breast cancer metastases versus paired primary would be intrinsic to cancer cells and not due to sample biopsy composition. To help sift through the top differential metastasis genes (Fig. 1a), we turned to the Cancer Dependency Map (DepMap) CRISPR assays [17, 21] measuring the essentiality of each gene for each of 46 breast cancer cell lines. A low DepMap-based gene effect score for a given gene in a cell line indicated that the cell line is dependent on the gene for proliferation in vitro. We overlapped the 2115 genes higher (*p* < 0.001, paired t-test) in the breast cancer metastasis signature (from Fig. 1a) with the set of 1810 genes that were essential (with gene effect score < − 0.75) in > 10% of breast cancer cell lines by DepMap. The overlap of 264 genes between the two result sets (Fig. 2a and Data File S1) was highly statistically significant (enrichment *p* < 1E−29, chi-square test, chance expected overlap of 136 genes) and represented an “essential” metastasis signature as explored further below. We observed no significant overlap between the genes lower in metastasis and genes essential by DepMap (Fig. 2a).

The set of 1581 genes higher in metastasis but for which no breast cell lines had low DepMap-based gene effect scores represented a “nonessential” metastasis signature that could serve as an interesting comparison and contrast to the essential metastasis signature of 264 genes (Figs. 2b and c). The remaining 270 metastasis genes with low gene effect scores but for fewer cell lines (Data Files S1) might also include genes of interest that would be intrinsic to cancer cells, though we focused our study on the above 264 essential and 1581 nonessential genes, as these gene sets should represent a sharper contrast. Notably, in contrast to the 1581 nonessential genes, the 264 essential genes were not significantly enriched for genes lower in normal breast tissue by GTEx (Fig. 2b). In addition, while the nonessential genes showed the above-noted association with liver biopsy site, the differential expression patterns for the 264 essential genes were much more consistent across diverse metastatic biopsy sites (Fig. 2c and d). Also, analysis of single cell RNA-sequencing (scRNA-seq) data from both metastatic and primary tumors showed the essential signature genes to be highly expressed as a group within the cancer epithelial cells (Supplementary Fig. 1a–c). Primary cancer cells scored moderate to high for the essential signature, though spatial transcriptomics did not reveal any clear patterns of the high metastasis signature cells as being on the invasive front of the tumor (Supplementary Fig. 1d). While genes with low DepMap gene effect scores in > 10% of cell lines were highly enriched for cell cycle genes, for example involving 651 genes by Whitfield et al. [22] (*p* < 1E−60, one-sided Fisher’s exact test, Data File S1), the essential metastasis signature genes, incorporating paired metastasis vs primary comparisons, were not similarly enriched for cell cycle genes (*p* = 0.06).

### The essential metastasis signature is manifested in primary breast tumors

**Fig. 3:**
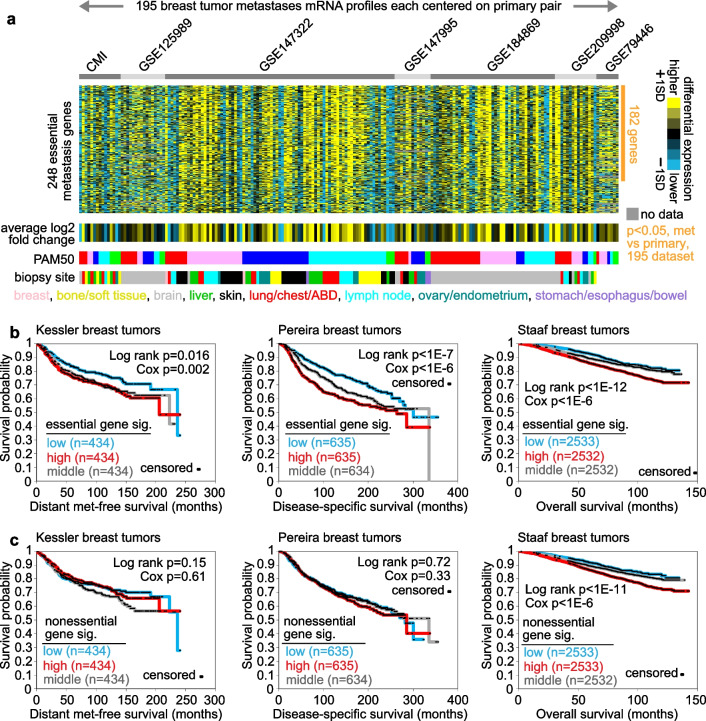
The essential metastasis signature patterns as examined in external breast cancer datasets. **a** For the essential metastasis genes from the BIG dataset (from Fig. 2a), heat map of differential expression in a compendium dataset of 195 breast metastases versus paired primary, representing seven individual studies [5–11]. Each metastasis expression profile in the compendium dataset was centered on its primary pair (not shown). Yellow, high expression in metastasis versus primary; blue, low expression in metastasis. SD, standard deviations from the centered metastasis and primary profiles within a given dataset. Of the 248 essential genes represented in the compendium dataset, 182 were differential expressed in metastasis versus primary tumors across the compendium (*p* < 0.05, paired t-test), a highly significant overlap (*p* < 1E−80, one-sided Fisher’s exact test). **b** Association of the metastasis essential gene signature with breast cancer patient survival across three separate expression datasets of primary breast tumors [27–29]. For each dataset, a gene signature score was derived using our “t-score” metric [52–54], comparing the average of the normalized expression values for the signature genes against the rest of the normalized gene values within the tumor profile. The Staaf et al. dataset represents early stage breast tumors [29]. P-values for association of signature score with patient outcome by log-rank test and by univariate Cox, as indicated. **c** Similar to part b, but using the nonessential signature genes (Fig. 2b) to compute a nonessential gene signature score in primary breast tumors

We could confirm the metastasis-specific expression patterns of most essential genes in an external compendium dataset of 195 breast metastases versus paired primary, representing seven individual studies [5–11]. Of the 248 essential genes represented in the compendium dataset, 182—73%—were differential expressed in metastasis versus primary tumors across the compendium (*p* < 0.05, paired t-test, Fig. 3a), a highly significant overlap (*p* < 1E−80, one-sided Fisher’s exact test). Notably, a smaller fraction of the nonessential genes—57%—were similarly significant in the compendium dataset (Data File S1). We also examined two gene signatures of breast cancer metastasis versus primary tumors derived from previous studies, one from Siegel et al. [23] and one from Chen et al. [24] (Data Files S1). Of the 123 genes high in the Siegel signature, 2 and 26 were in our essential and nonessential metastasis gene sets, respectively, the latter representing a significant overlap (*p* < 1E−9, one-sided Fisher’s exact test). Similarly, of the 337 genes high in the Chen signature, 0 and 147 were in our essential and nonessential metastasis gene sets, respectively (*p* < 1E−90 for the 147 genes).

Results of previous studies have suggested that the metastatic potential of human tumors is encoded within the primary tumor [25, 26]. We applied our essential metastasis gene signature to each of three independent gene expression datasets of primary breast tumors [27–29]. For each dataset, the top set of genes associated with worse patient prognosis (*p* < 0.01 by univariate Cox) shared significant overlap with the essential metastasis signature genes (*p* < 1E−25, by one-sided Fisher’s exact test, for each dataset), while better prognosis genes were anti-enriched for the essential signature genes (Data File S1). For each dataset, we computed a signature score derived from the essential genes. Based on the expression patterns of primary tumors, the essential metastasis signature could stratify patients in each dataset into high-, low-, and intermediate-risk groups representing significant differences in patient outcome by Kaplan–Meier analysis (Fig. 3b). The signature was also associated with worse patient outcome by univariate Cox, which treated the signature score as a continuous variable without grouping patients. These results included the “Scan-B” expression dataset from Staaf et al. consisting of 7598 early stage breast cancers [29]. Interestingly, the nonessential metastasis signature was associated with worse breast cancer patient outcome in only one of the three primary tumor datasets (Fig. 3c). In multivariate Cox models incorporating mRNA expression of proliferation gene marker MKI67 with the essential metastasis signature score, the latter remained statistically significant, indicative of its representing additional information from cell proliferation (two-sided *p* < 0.0001 and *p* < 1E−6 for Pereira [28] and Staaf [29] datasets, respectively; one-sided *p* = 0.046 for Kessler [27] dataset). However, in multivariate Cox models incorporating other well-known prognostic signatures [26, 30] in addition to the essential metastasis signature, the latter was found to not provide additional prognostic information in two out of three datasets (Table S1).

### Pathways and functional gene groups represented by the metastasis signature

**Fig. 4:**
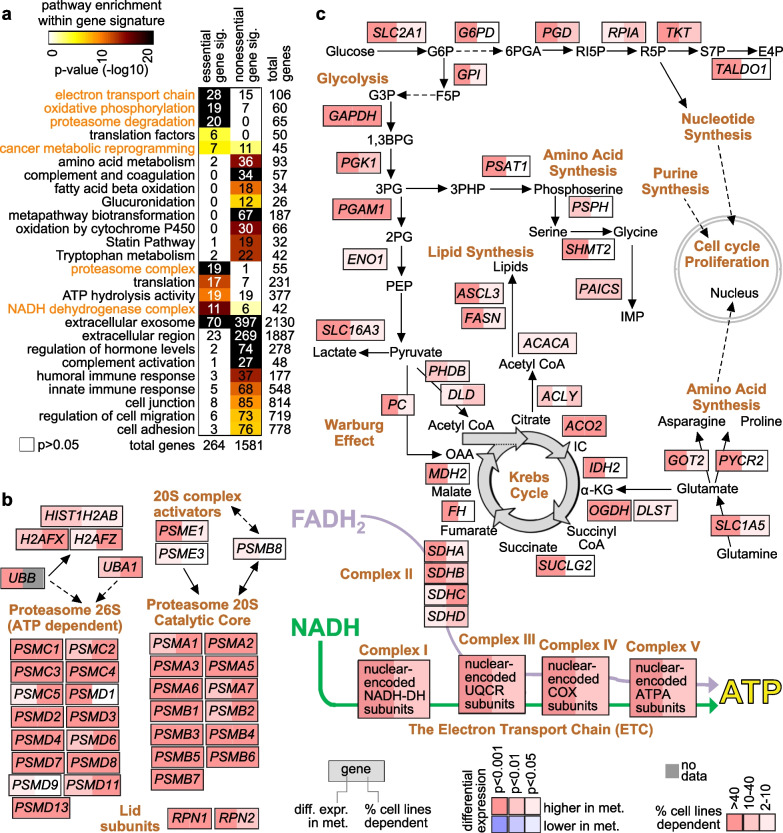
Pathways associated with the essential breast cancer metastasis signature. **a** Selected significantly enriched Gene Ontology (GO) terms [31] and wikiPathway [32] gene sets involving either the essential or nonessential set of metastasis-associated genes. Enrichment p-values by one-sided Fisher’s exact test. Some wikiPathways are slightly abbreviated, e.g., “Metabolic reprogramming in colon cancer,” abbreviated as “cancer metabolic reprogramming.” Pathways and gene sets highlighted in orange are featured in the pathway diagrams of parts b and c. **b** Pathway diagram representing genes involved in proteasome degradation [32]. On the left of each gene is represented the significance of differential expression in breast metastasis versus paired primary (based on BIG dataset; red, higher in metastases). On the right of each gene is represented the percentage of breast cancer cell lines in the DepMap dataset with low gene effect scores (< − 0.75). **c** Similar to part b, but featuring a pathway diagram representing core metabolic pathways [32, 55, 56]

The essential metastasis signature genes represented functional gene categories and altered pathways (Fig. 4a and Data File S2). In terms of functional gene categories, we found significantly enriched Gene Ontology (GO) annotation terms [31] (representing gene annotation by molecular function, biological process, and cellular component) for the essential genes, including ‘proteasome complex’, ‘translation’, ‘ATP hydrolysis activity’, ‘NADH dehydrogenase complex’, and ‘extracellular exosome’. Enriched wikiPathway [32] gene sets (representing manually curated pathways) for the essential genes included several consistent with the GO terms results and included pathways related to the electron transport chain, oxidative phosphorylation, proteasome degradation, translation factors, and cancer metabolic reprogramming. In contrast, the nonessential metastasis signature genes were significantly enriched for genes related to extracellular region, cell junction, cell adhesion, and the immune response, consistent with these genes representing non-breast tissue markers as well as complement pathway genes produced by the liver [33] (Data Files S1 and S2). Both 20S and 26s proteasomes included several genes both higher in breast cancer expression in metastasis and essential in more than 90% of breast cancer cell lines in DepMap (Fig. 4b and Data File S1). When surveying core metabolic pathways (Fig. 4c), genes both higher in expression in metastasis and essential in one or more breast cancer cell lines included glycolysis, lipid synthesis, the Krebs cycle, the Warburg effect, and the electron transport chain. The essential metastasis genes involving the electron transport chain spanned complexes I–V (Fig. 4c and Data Files S1 and S2). GO terms and pathways involving the cell cycle were not significantly enriched in either metastasis gene set (Data File S2).

### Transcription factor (TF) and global targeting associations

**Fig. 5:**
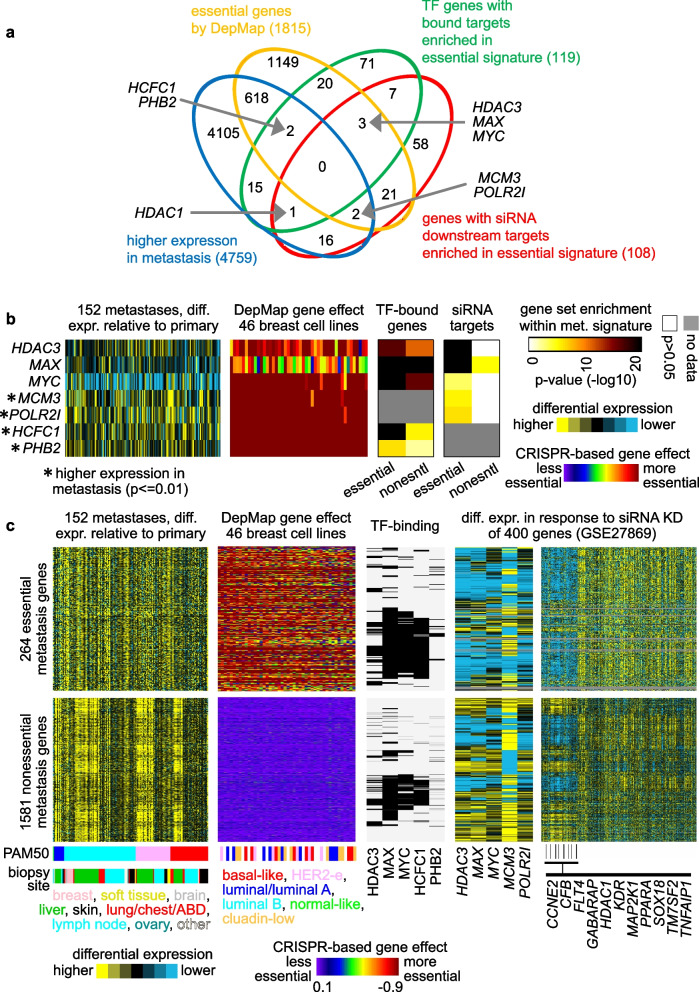
Transcription factor (TF) and global targeting associations involving the essential metastasis signature. **a** Venn diagram of the overlapping genes involving four gene sets: genes with high expression in breast cancer metastasis versus paired primary (blue, based on BIG, using a relaxed p-value of 4759 by paired t-test); genes essential in at least 10% of breast cancer cell lines (light orange, using effect score < − 0.75); TF genes with bound targets enriched (*p* < 0.01, one-sided Fisher’s exact test) in the essential gene signature (green, TF associations by Encode [34] data); and genes for which siRNA knockdown results in under-expression of genes that are significantly enriched (*p* < 0.01, one-sided Fisher’s exact test) in the essential metastasis signature (red, using a gene expression dataset of human umbilical endothelial cells transfected with siRNAs for 400 different genes [35]). **b** For the seven genes overlapping among three of the four gene sets from part a, the corresponding patterns are represented involving differential expression in breast cancer metastasis, CRISPR-based gene effect scoring in breast cancer cell lines, enrichment of TF-bound genes by Encode data, and enrichment of siRNA targets. Gene set enrichment patterns (by one-sided Fisher’s exact test) are represented for both essential and nonessential metastasis gene sets. **c** For both essential and nonessential metastasis signature genes (top and bottom, respectively), the corresponding patterns are represented involving differential expression in metastases, CRISPR-based gene effect scoring, binding 2 kb upstream for selected TFs, and differential expression in response to siRNA knockdown (GSE27869 dataset [35] involving profiling of cells after knockdown of each of 400 genes)

To gain additional insights into possible drivers underlying the essential gene signature of metastasis, we turned to two additional orthogonal datasets: one of TF-bound target genes cataloged by the Encode project [34] and another of cells profiled for gene expression after siRNA knockdown for each of 400 different genes [35]. Out of 158 TFs with available data [6], 119 were significantly enriched (*p* < 0.01, one-sided Fisher’s exact test) for essential metastasis signature genes (Data File S2). Of the 400 genes knocked down in the siRNA expression dataset, 108 had genes that were under-expressed with their knockdown (the under-expressed genes representing downstream targets of the knocked down gene) being significantly enriched (*p* < 0.01) for essential metastasis signature genes (Data File S1). We intersected the above 119 and 108 genes with the genes essential in breast cancer cell lines and the genes more highly expressed in breast cancer metastases (using more relaxed criteria for differential expression to include more genes). No genes were found within all four gene sets, but several genes of interest involved three of the four gene sets (Figs. 5a and b). Genes *HCFC1* and *PHB2* were higher in breast cancer metastases versus paired primary, essential in all 46 breast cancer cell lines, and encoded TFs for which their bound targets involved over-representation of our essential metastasis signature genes. *MCM3* and *POLR2I* were higher in metastases, essential, and had their downstream targets (by RNAi) enriched for our essential metastasis signature genes. Genes *HDAC3*, *MAX*, and *MYC* were all essential for many cell lines and encoded TFs with both bound and downstream targets enriched for our essential metastasis signature genes.

We examined both essential and nonessential metastasis signature genes across all the above datasets, including differential expression patterns in metastasis, gene target binding for selected TFs, and differential expression patterns in response to the knockdown of selected genes (Fig. 5c). Notably, TF-bound targets of MYC, MAX, and HCF1 overlapped highly with each other, with these bound targets also overlapping genes with lower expression with *MYC* or *MAX* knockdown. Of note, MAX is essential for MYC’s oncogenic function in its dimerization with MYC [36, 37], and MYC is known to directly interact with a recruits HCFC1 [36], where the MYC-HCFC1 interaction in particular can regulate mitochondrial gene expression programs among others [36, 38, 39]. The nonessential signature genes were also significantly enriched for targets of TFs, including MYC, MAX, and HCFC1, though these three TFs involved a relatively lower fraction of genes for the nonessential versus the essential signature (Fig. 5c and Data File S2). Interestingly, where the nonessential genes overlapped with the above TF-related and knockdown target-related patterns of interest, these genes showed a weaker association with liver biopsy site than that of the other nonessential genes (Fig. 5c). Genes such as *MYC* and *MAX* that did not have relatively higher expression in metastases at the mRNA level could still be active at the signaling level.

## Discussion

In this study, we identified an essential gene signature of breast cancer metastasis, defined as the intersection of genes with higher expression in metastasis versus paired primary and genes essential in breast cancer cell lines by CRISPR assays. We found a significant overlap between these two orthogonal gene sets, indicative of true biological changes intrinsic to cancer cells involving a fraction of the global differences within the metastasis expression profile. While the essential metastasis signature genes were comprised of a subset of genes essential for breast cancer cell survival, the signature represented more than simply a generic signature of cell proliferation, as, for example, genes involved in the cell cycle, while essential in breast cancer cells, tended not to be higher in metastasis versus paired primary. The nonessential metastasis genes, differentially higher in expression in metastasis but not essential in any breast cancer cell lines, provided a clear contrast to the essential metastasis genes, further establishing the essential genes as underscoring metastasis biology. In contrast to nonessential metastasis genes, essential metastasis genes: did not globally represent non-breast versus breast tissue expression differences, did not show strong associations with tissue biopsy site, had a greater percentage of genes with confirmed higher expression in metastasis in an external compendium dataset, had more dramatic associations with patient outcome based on expression in primary tumors, and involved an entirely different set of enriched functional gene sets and pathways. Transcription factor genes that appear to drive the expression of a sizable portion of the essential metastasis signature genes include *MYC*, *MAX*, *HDAC3*, and *HCFC1*.

Our study shows the need to refine gene expression differences between metastasis and primary tumors to identify better the metastasis-associated genes likely to play a role in cancer biology. Previous studies have utilized gene expression data of breast cancer metastases with paired primary to identify patterns of subtype switching and have utilized annotated gene signatures to examine differences corresponding to cell type, including immune cell types [4, 5]. Here, we used a different approach to incorporate functional data from breast cancer cell lines to refine the global signatures for the genes essential for proliferation in vitro. While most cell lines have undergone numerous passages, and their molecular state may be far removed from that of the original cancer from whence they came, they are quite amenable to functional interrogation, would not have the issue of non-cancer cell admixture involved in human biopsies, and offer public omics data resources that can readily be leveraged in the interpretation of results from human tumors [6, 40]. From a practical standpoint, our approach effectively established a metastasis signature for which non-cancer cell type patterns were not dominant. Furthermore, the overlap between the cell line and metastasis results was highly statistically significant, with the overlapping genes indicative of a non-random relationship between the two results sets. At the same time, many genes essential to breast cancer cell proliferation would not just be essential to those cells but also for cells in other tissues. Other analytical approaches to refine gene signatures of metastases might be explored in future studies. For example, it might be possible to computationally deconvolute the different cell types within a bulk metastasis expression profile [41], though doing so with high confidence might require precise information as to the different cell types involved and their fractional contribution.

Our essential signature of breast cancer metastasis could have important implications for applying existing therapies or developing alternate therapeutic approaches. In recent years, compounds directly or indirectly inhibiting MYC have shown anticancer activity preclinically, with some of these being developed for clinical trial evaluation [42, 43], and MYC inhibition has been found to halt metastatic breast cancer progression by blocking growth, invasion, and seeding [44]. Proteasome inhibitors, such as bortezomib and carfilzomib, are highly effective in treating solid tumors, and proteasome inhibitors have been found to re-sensitize the standard chemotherapeutic regimens and induce synergistic anticancer effects in breast cancer [45]. Bortezomib has been shown to inhibit breast cancer growth and reduce osteolysis by downregulating metastatic genes [46]. There has been accumulating evidence suggesting that the glycolytic pathway is upregulated in various cancer types and is responsible for their aggressive phenotype, consistent with the concept of the “Warburg effect,” whereby tumor cells predominantly utilize energy through high rates of glycolysis [47, 48]. The reliance of metastatic cells on mitochondrial respiration and oxidative phosphorylation can be exploited using drugs that target mitochondrial metabolism, including therapeutic agents that activate signaling pathways that promote the production of reactive oxygen species (ROS), a reduction in antioxidant defenses, or both [49].

Additional genes and pathways involved in breast cancer metastases would remain to be discovered in future studies. Our essential metastasis signature gene patterns would span multiple intrinsic breast cancer subtypes, though changes specific to a particular subtype might be discoverable [6]. New information on molecular pathways could reveal their enrichment within metastasis signature genes. As more data become available, pan-cancer signatures of metastases could shed light on molecular mechanisms spanning diverse tissues of primary origin in addition to breast. Our essential metastasis signature focuses on genes essential to cancer cell proliferation, though other genes not essential may conceivably have a role in the metastatic process. For example, our results would not represent tumor microenvironmental changes that might influence metastasis. Transcriptional programs driving organ-specific patterns of metastasis would be discoverable by combining patient molecular data with molecular data from experimental models [50]. Future scRNA-seq studies profiling large numbers of paired metastatic and primary tumors could identify changes occurring within isolated tumor cell populations. Signaling changes at the protein or signal transduction levels would not necessarily be reflected in the metastatic cancer transcriptome, though these may be uncovered using other omics platforms. Given the metabolic pathway associations uncovered here in metastatic transcriptional programs, it would be worthwhile to generate metabolomic profiles of paired metastases and primary tumors at a large scale [51]. Nevertheless, our present study sheds light on a gene set of high interest regarding breast cancer metastasis, with these genes collectively associating with robust patterns involving pathways and driver genes that would merit further exploration.

## Methods

### BIG breast cancer metastases dataset

Regarding human subjects, cancer molecular profiling data used in the present study were generated through informed consent as part of previously published studies and analyzed in accordance with each original study’s data use guidelines and restrictions.

BIG conducted Aiming to Understand the Molecular Aberrations in Metastatic Breast Cancer (AURORA; NCT02102165), a molecular screening program that involved extensive profiling of paired primary breast tumors and metastatic samples [4]. After entering into a data access agreement with BIG, we obtained the RNA-seq dataset from the AURORA study [4] representing 314 breast cancer patients, of which 152 had both a metastasis with paired primary profiled for gene expression. Taking the entire processed RNA-seq dataset of transcripts per million (TPM) expression values from 466 tumor profiles (314 patients), we first carried out quantile normalization [58] (limiting the dataset to genes with Entrez identifier) and then log2-transformed values. We then compared metastasis versus primary in a paired analysis using a paired t-test across the 152 patients with paired samples. The BIG gene expression dataset represented 28,248 uniquely identified genes (by Entrez) with detected expression allowing for paired primary-metastasis comparisons. The numbers of differentially expressed genes at a nominal *p* < 0.001 far exceeded the chance expected (~ 28 versus 3927 actual genes, FDR = 0.007) that would be due to multiple gene testing [59]. When overlapping different top-gene results sets (e.g., overlapping genes higher in metastasis with TF bound genes or siRNA-targeted genes), we used a more relaxed p-value cutoff for differential expression to limit false negatives, helping us identify significant overlap patterns.

### GTEx normal tissue expression dataset

Gene expression data (TPM values) from GTEx Analysis version 7 release were obtained from the GTEx Portal (https://www.gtexportal.org) [19]. Using log-transformed values, we compared normal breast tissues (n = 290 individuals) with normal non-breast tissues (n = 11,398 samples in all) by t-test. Non-breast tissues included adipose tissue, adrenal gland, blood vessel, bladder, brain, blood, skin, cervix uteri, colon, esophagus, fallopian tube, heart, kidney, liver, lung, salivary gland, muscle, nerve, ovary, pancreas, pituitary, prostate, small intestine, spleen, stomach, testis, thyroid, uterus, and vagina.

### Essential genes by the cancer Dependency Map

We examined gene effect scores (with low scores denoting essential genes) based on Cancer Dependency Map (DepMap) CRISPR assays, using the dataset as analyzed using the Chronos algorithm from Dempster et al. [17] We focused on the 46 breast cancer cell lines with DepMap data, and we used a cutoff score of < − 0.75 to denote gene essentiality in a given cell line. The breast cell line PAM50 subtype, as available, was taken from the annotation by Heiser et al. [60].

### External breast tumor expression compendium dataset

From public datasets external to the BIG dataset, we assembled a compendium dataset of gene expression profiling data of breast patient paired metastases and corresponding primary tumor. This compendium represented 195 patient metastases and seven individual studies [5–11] (Data File S1). We obtained processed expression data tables from the Gene Expression Omnibus (GEO) or the Genome Data Commons (in the case of the Count Me In or CMI dataset). To normalize the metastasis profiles relative to the paired primary, we first centered log2-transformed expression values for each metastasis expression profile on its primary pair, setting the values for the primary pair to zero. Then, within each study dataset, the centered expression values were divided by the standard deviation across the centered metastasis and primary profiles. This normalization step rendered the differential expression values unitless, thereby correcting for inter-dataset differences. Of the 195 metastases, 159 were represented in a metastasis expression compendium in our previous study [6], and here we added sample profiles from the following additional datasets: GSE79446, GSE147995, and GSE209998. The compendium dataset represented 18,319 genes [6]. For GSE209998, we incorporated only the sample profiles for which both the metastasis and the paired primary were both from fresh frozen samples (with other tumor metastases in that dataset involving either the metastasis or paired primary or both being sampled from Formalin-Fixed, Paraffin-Embedded (FFPE) tissue blocks.

The PAM50 subtype of each metastasis and primary sample was determined using either the original study annotation, where available, or inferred by the global expression profile in the following manner. We took the breast tumor expression dataset from Hoadley et al. [61], for which tumors were molecularly subtyped by PAM50 assay. For each gene common to our compendium and the Hoadley dataset, we computed the mean centroid of the four major Hoadley tumor subtypes and centered each group average on the centroid. With our compendium dataset, we centered the log2 expression values for the metastasis profiles and the primary tumors separately within each study to standard deviations from the median. We then took the Pearson's correlation (using all genes common to both data sets) between the Hoadley centered averages and the expression values of each tumor profile, and the subtype with centroid having the highest correlation was assigned to the sample profile.

### Survival analyses in primary breast tumors

We examined metastasis gene sets in public transcriptomic datasets of primary breast tumors for associations between the gene set and patient outcome. We referred to three datasets from Kessler et al. [27], Pereira et al. [28], and Staaf et al. [29]. The Kessler expression dataset represented a compendium involving 1302 patients and nine separate datasets assembled previously [27, 62]. We originally downloaded the Pereira et al. expression dataset from CBioPortal. The Staaf et al. dataset was obtained from the Mendeley Data site associated with the publication. Given a gene signature, we scored tumor expression profiles in the external breast cancer datasets using our previously described “t-score” metric [52–54]. With the primary breast tumor expression profiles normalized gene-wise to standard deviations from the median, we compared within each profile the average of the normalized expression values for the signature genes against the rest of the normalized gene values. The gene signature t-score is defined here as the two-sided t-statistic comparing the metastasis-associated genes with all other genes. We assessed the association of this gene signature score with patient outcome using univariate Cox and log-rank (dividing the cases according to low, high, or intermediate signature scoring). We used the same t-score metric in scoring the primary breast tumor expression datasets for two other well-known prognostic signatures (but here with up signature genes being compared with down signature genes) [26, 30].

By unsupervised clustering approaches, most gene signatures can be shown to associate with breast cancer patient outcome [63]. In contrast to unsupervised clustering approaches such as principal components analysis, our t-score is supervised in that it imposes a pre-defined direction upon the genes in the signature. In our present study, tumors that scored highly by t-score metric would have all the signature genes higher as a group on average than the other genes in the tumor profile. In addition, using the Pereira dataset, we carried out an exercise with 100 randomly generated gene signatures of the same size as the essential gene signature. Across all the expression profiles in the Pereira dataset, the standard deviation of the scores for the actual signature was higher than that of the random signatures, indicative of a greater level of coordinate expression of the essential metastasis signature genes across tumors. When assessing the association of the random gene signatures with patient outcome, only one of the 100 signatures had a univariate Cox p-value smaller than the actual essential signature.

### Enrichment analyses for TF-bound genes

We obtained TF binding site locations based on ENCODE consortium data from chromatin immunoprecipitation sequencing (ChIP-seq) [34], from Ensembl (GRCh37/hg19 build). We used TF sites as identified in the HeLa-S3, HepG2, and K562 cell lines (accessed April 2022), involving 158 TFs. We defined associations between TFs and genes as a TF binding site falling within 2 kb upstream of the gene start. For each TF and each PDX-based subtype, we identified patterns of significant gene set overlap (by one-sided Fisher's exact test) between the TF-bound genes and the genes in the given set of interest.

### Gene targets of siRNA knockdown

For expression alterations in response to gene knockdown, we referred to the GSE27869 expression profile dataset of human umbilical vein endothelial cells (HUVECs) transfected with siRNAs for 400 different genes [35]. We normalized log2 gene expression values in GSE27869 to standard deviations from the median across the 400 profiles. Of the 400 genes represented in GSE27869, 44 involved the 158 TFs surveyed using Encode data (see above). For each siRNA differential expression profile, we took the set of genes under-expressed with normalized expression < − 0.5. We assessed the enrichment of metastasis gene sets within the siRNA-associated under-expressed genes using one-sided Fisher’s exact tests.

### Analysis of single cell RNA sequencing (scRNA-seq) and spatial transcriptomics data

We obtained scRNA-seq data for two breast tumors from two separate studies: Slyper et al. [57] of a metastatic breast cancer (MBC) sample (GSM4186971) and Wu et al. [16] of a primary triple negative breast tumor (TNBC, sample CID44971). For scoring cell expression profiles for a given gene signature, we first filtered the geneXcell counts matrices for genes with > 10% nonzero values across cells, then imputed the median value across cells for each matrix entry of zero, then quantile normalized the imputed counts dataset [58]. We log2-transformed the normalized counts data, centered each gene across cells to standard deviations from the median, and then took the average of the signature genes (essential and nonessential signatures) within each normalized cell profile as the gene signature score for that cell. We analyzed scRNA-seq and spatial transcriptomics data using the Seurat package [64].

### Statistical analysis

All p-values were two-sided unless otherwise specified. We evaluated the enrichment of GO annotation terms [31] and wikiPathways [32] within sets of differentially expressed genes was evaluated using SigTerms software [65] and one-sided Fisher’s exact tests. Visualization using heat maps was performed using both JavaTreeview (version 1.1.6r4) [66] and matrix2png (version 1.2.1) [67]. Figures indicate exact the value of n (number of tumors or cell lines), and the statistical tests used are noted in the Figure legends and next to reported p-values in the Results section. Boxplots represent 5%, 25%, 50%, 75%, and 95%. Figures represent biological and not technical replicates.

### Supplementary Information

Supplementary Table 1.

Supplementary Fig. 1. Analysis of the essential metastasis signature in single cell RNA-sequencing (scRNA-seq) and spatial transcriptomic datasets. scRNA-seq data for two breast tumors from two separate studies are presented here: one from Slyper et al.[57] of a metastatic breast cancer (MBC) sample (GSM4186971) and one from Wu et al.[16] of a primary triple negative breast tumor (TNBC, sample CID44971). **(a)** UMAP plots showing major cell populations identified from the MBC (left) and the TNBC (right). **(b)** Using the counts matrix, we scored each cell profile for the essential gene signature (based on the average normalized expression of genes in the signature). Boxplots represent the essential gene signature scoring by cell type. For the MBC sample (left), the epithelial cell group shows dramatically higher levels of the essential gene signature as compared to the non-epithelial cell types, consistent with our notion that the essential gene signature would be mostly representative of the metastatic cancer cells versus the non-cancer cells comprising the sample biopsy. The essential signature also appears elevated in the Ductal carcinoma in situ (DCIS) cells of the primary TNBC sample (right), again consistent with the notion of the signature patterns being intrinsic to cancer cells, as well as the notion that the metastasis signature may also be present and at work within primary tumor cells (e.g., as also indicated in main Figs. 3b and 3c). **(c)** Similar to part b, but for the nonessential metastasis signature. Interestingly, in the MBC sample, the nonessential signature scoring appears markedly higher for macrophages as compared to the essential signature, where we expect the nonessential signature to represent more of the “noise” in the BIG AURORA data. At the same time, epithelial cells in the MBC have the highest levels of the nonessential signature, where many bona fide metastasis-intrinsic genes could still be present in the nonessential signature (while being enriched for non-specific genes, e.g., as suggested by Fig. 5c). For the primary TNBC, the nonessential signature is also elevated in the DCIS cells. For parts b and c, boxplots represent 5%, 25%, 50%, 75%, and 95%. **(d)** Spatial data from the Wu et al.[16] study for the TNBC sample CID44971. Left, cells in the tumor are colored according to cell type. Right, cells in the tumor are colored according to scoring for the essential metastasis signature. Most DCIS cells score moderate to high for the signature.

Data File S1. Gene-level correlations and tumor-level sample annotation. Provided as an Excel file. For 28,248 unique genes (by Entrez identifier) represented in the BIG breast cancer metastasis dataset, paired metastasis versus primary tumor statistics are provided, along with other gene-level information used in the study (DepMap, GTEx, etc.). Also included are sample information for the profiles analyzed for the BIG breast cancer metastasis dataset and the compendium dataset of 195 breast metastases versus paired primary, representing seven individual studies. The corresponding data for the 264 essential metastasis signature genes are provided for DepMap breast cell lines and for differential expression in response to siRNA knockdown in the GSE27869 dataset.

Data File S2. Pathway and gene set enrichment for the metastasis signature genes. Provided as an Excel file. For both essential and nonessential metastasis signatures, significant enrichment patterns involving GO terms, wikiPathways, and TF-bound genes are provided, along with the corresponding metastasis gene-to-gene set associations for the significantly enriched gene sets.

## Data Availability

Instructions to access the manuscript processed data are available at the webpage https://aurora.bigagainstbreastcancer.org/ and can be obtained upon signature of an appropriate data transfer agreement subject to applicable laws. Instructions to access processed or raw manuscript data to perform original research are also available on the webpage and investigators can contact aurora.researchproposals@bigagainstbc.org for enquiries. Access to data for research will be granted upon review of a project proposal and endorsement by the study Steering Committee, and after entering into an appropriate data access agreement subject to applicable laws.

## Abbreviations

BIG: Breast International Group
GTEx: Genotype-Tissue Expression Project
DepMap: The Cancer Dependency Map Project
CRISPR: Clustered regularly interspaced short palindromic repeats
RNA-seq: RNA sequencing
TF: Transcription factor
