# Supplementary material for: An essential gene signature of breast cancer metastasis reveals targetable pathways
Source: Breast Cancer Res. 2024 Jun 12;26:98. doi: 10.1186/s13058-024-01855-0 (PMC11167932; doi:10.1186/s13058-024-01855-0)
Supplement: Supplementary file 1 — Supplementary Table 1. [file 13058_2024_1855_MOESM1_ESM.pdf]

Table S1. Multivariate Cox incorporating multiple prognostic signatures.

	Coefficient	95 % Conf. (±)	Std.Error	P	Hazard = Exp(Coef.)
Kessler gene expression dataset					
essential	-0.037	0.129	0.066	0.57	0.964
70-gene	-0.017	0.177	0.09	0.85	0.983
GGI	0.378	0.204	0.104	0.00028	1.46
Pereira gene expression dataset					
essential	-0.028	0.105	0.053	0.6	0.973
70-gene	-0.053	0.154	0.078	0.5	0.949
GGI	0.401	0.188	0.096	0.000028	1.494
Staaf gene expression dataset					
essential	0.277	0.061	0.031	0.0000011	1.32
70-gene	-0.292	0.061	0.031	0.00000091	0.747
GGI	-0.096	0.06	0.031	0.0019	0.909

essential signature, the 264 genes featured in the present study; 70-gene signature from van't Veer et al. (PMID:11823860); GGI, Genomic Grade Index signature, from Loi et al. (PMID:17401012). For each signature, signature scores are normalized to standard deviations from the median. Adverse event for Kessler dataset is distant metastasis-free survival; for Pereira, disease-specific survival; for Staaf, overall survival.
