# Supplementary figures and images for: An essential gene signature of breast cancer metastasis reveals targetable pathways
Source: Breast Cancer Res. 2024 Jun 12;26:98. doi: 10.1186/s13058-024-01855-0 (PMC11167932; doi:10.1186/s13058-024-01855-0)

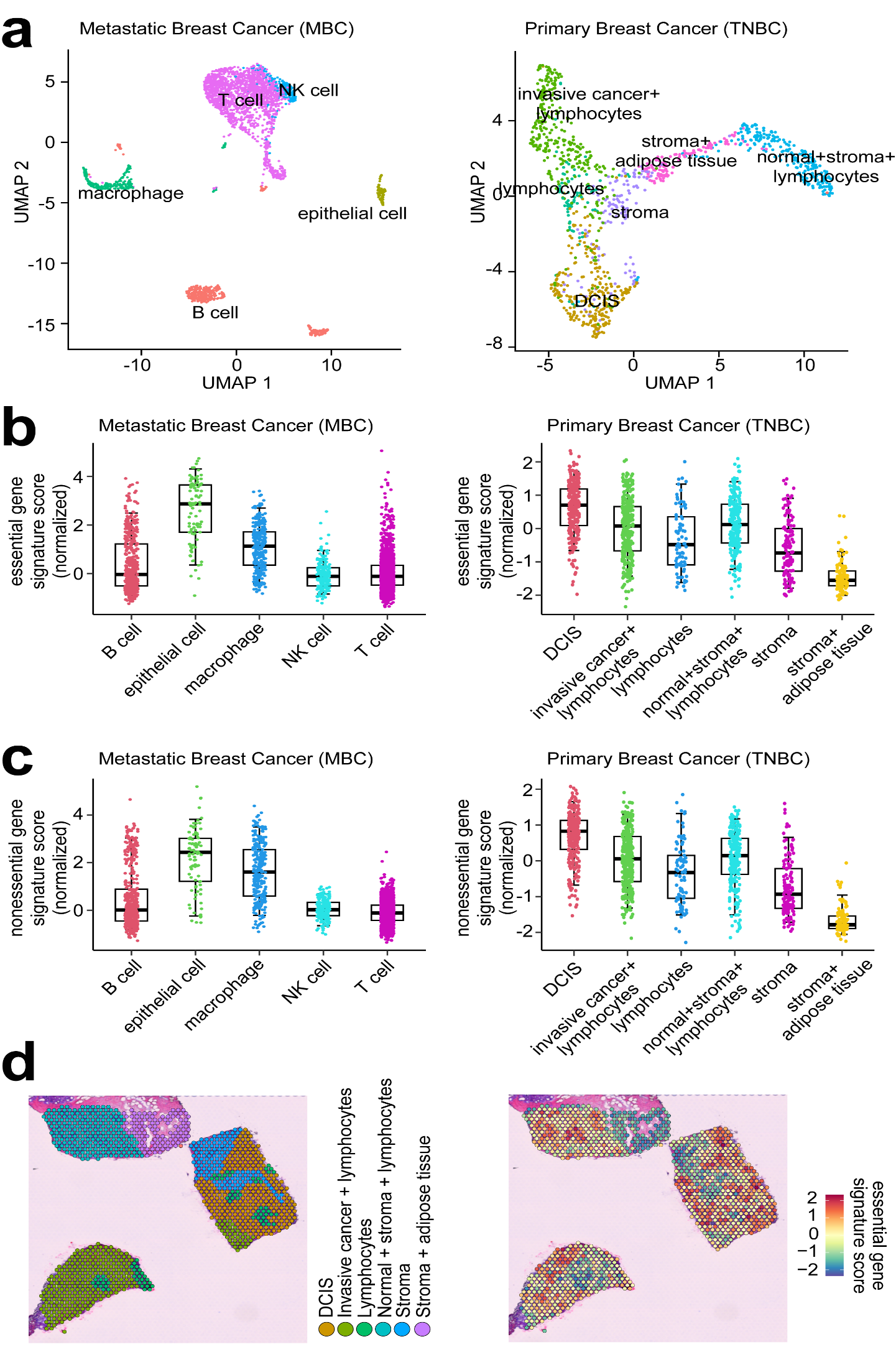

Supplement: Supplementary file 2 — Supplementary Fig. 1. Analysis of the essential metastasis signature in single cell RNA-sequencing (scRNA-seq) and spatial transcriptomic datasets. scRNA-seq data for two breast tumors from two separate studies are presented here: one from Slyper et al.[57] of a metastatic breast cancer (MBC) sample (GSM4186971) and one from Wu et al.[16] of a primary triple negative breast tumor (TNBC, sample CID44971). (a) UMAP plots showing major cell populations identified from the MBC (left) and the TNBC (right). (b) Using the counts matrix, we scored each cell profile for the essential gene signature (based on the average normalized expression of genes in the signature). Boxplots represent the essential gene signature scoring by cell type. For the MBC sample (left), the epithelial cell group shows dramatically higher levels of the essential gene signature as compared to the non-epithelial cell types, consistent with our notion that the essential gene signature would be mostly representative of the metastatic cancer cells versus the non-cancer cells comprising the sample biopsy. The essential signature also appears elevated in the Ductal carcinoma in situ (DCIS) cells of the primary TNBC sample (right), again consistent with the notion of the signature patterns being intrinsic to cancer cells, as well as the notion that the metastasis signature may also be present and at work within primary tumor cells (e.g., as also indicated in main Figs. 3b and 3c). (c) Similar to part b, but for the nonessential metastasis signature. Interestingly, in the MBC sample, the nonessential signature scoring appears markedly higher for macrophages as compared to the essential signature, where we expect the nonessential signature to represent more of the “noise” in the BIG AURORA data. At the same time, epithelial cells in the MBC have the highest levels of the nonessential signature, where many bona fide metastasis-intrinsic genes could still be present in the nonessential signature (while being e [file 13058_2024_1855_MOESM2_ESM.docx]
